# Potential ecological risk of heavy metals in sediments from the Mediterranean coast, Egypt

**DOI:** 10.1186/s40201-015-0223-x

**Published:** 2015-10-10

**Authors:** Naglaa Farag Soliman, Samir Mahmoud Nasr, Mohamed Abdelaziz Okbah

**Affiliations:** Department of Environmental Studies, Institute of Graduate studies and Research, Alexandria University, Alexandria, Egypt; Department of Marine Chemistry, National Institute of Oceanography and Fisheries, Alexandria, Egypt

**Keywords:** Heavy metals, Potential Ecological risk index, Sediments, Mediterranean coast, Egypt

## Abstract

**Background and aim:**

Mediterranean Sea, Egypt is an economically important marine environment. During the last decades there has been extensive increase in the levels of urbanization and industrialization along its coastal area. Therefore, the present work attempts to determine the status of heavy metals distribution in sediment samples, and their ecological risk assessment in the studied area.

**Materials and methods:**

Twenty surfacial sediment samples were collected from different selected stations along the Egyptian Mediterranean Sea. The samples were homogenized and placed into sealed polyethylene bags, carried to the laboratory in an ice box and stored at −20 °C in the dark until analysis.

**Results:**

The results revealed that Fe had the highest mean value (243–38045 μgg^−1^) followed by Mn (17–1086 μgg^−1^), and a lower concentrations were found for Co (0.43–26.39 μgg^−1^) and Cd (0.04–0.47 μgg^−1^). Risk assessment showed that Cd had the highest ecological risk (Er = 21.52), followed by Pb (Er = 3.01), while Zn had the lowest risk (Er = 0.23). Both the ecotoxicological index method and the potential ecological risk index (RI) suggested that the combined ecological risk of the studied metals may be low. Multivariate statistical analysis (Cluster and Factor analysis) suggested that the lithogenic factor dominants the distribution of most part of the considered metals in the study area.

**Conclusion:**

Multivariate analysis has been proved to be an effective tool for providing suggestive information regarding heavy metal sources and pathways. The results of this study provide valuable information about metal contamination in sediments along the Mediterranean Sea for over than 1200 km.

## Introduction

Heavy metals pollution of aquatic environment has become a great concern in recent years [1]. Heavy metals are among the most persistent of pollutants in the ecosystem such as water, sediments and biota because of their resistance to decomposition in natural condition. Toxicity appears after exceeding level of indispensability. Heavy metals become toxic when they are not metabolized by the body and accumulate in the soft tissues [2]. Under certain environmental conditions, heavy metals might accumulate up to toxic concentrations levels, and cause ecological damage [3]. Iron, zinc, copper and manganese are essential metals since they play important roles in biological systems [4], but they become toxic at higher concentrations. Non-essential metals such as Pb, Cd are usually potent toxins even at relatively low concentrations and their bioaccumulation in tissues leads to intoxication, cellular and tissue damage, decreased fertility, dysfunction of a variety of organs and cell death [5]. Lead, cadmium, have been included in the regulations of the European Union for hazardous metals [6], while chromium and nickel are listed as hazardous metals by the United States Food and Drug Administration (USFDA) [7]. Metals have low solubility in water, get adsorbed and accumulated on bottom sediments [2]. While metals settled in sediments may be re-suspended and cause secondary contamination to the water environment, because sediments act both as a sink and a source for metals in the aquatic environment [8]. This fact converts the sediments in a permanent record of anthropogenic pollutants inputs [9]. Therefore, spatial surveys of metal concentrations in the sediments and then comparisons with non-polluted baselines are important to understand the mechanisms of accumulation and geochemical distribution of heavy metals in the aquatic systems and to provide basic information for the judgment of environmental health risks [10].

To date, many methodologies have been developed to assess ecological risks of heavy metals. However, most of them are suitable only for ecological assessment of a single contaminant (e.g., Geoaccumulation index method and Enrichment factor). In reality, many kinds of heavy metals usually accumulate simultaneously and cause combined pollution. To address this, Hakanson [11] developed the potential ecological risk index, which introduced a toxic-response factor for a given substance and thus can be used to evaluate the combined pollution risk to an ecological system [12]. On the other hand, mean SQG quotient (mSQGQs) has been developed for assessing the potential effects of contaminant mixtures in sediments. Mean SQGQ have been calculated most frequently with SQGs derived with empirical approaches, such as the ERM, PEL values, in which measures of adverse effects were associate with, but not necessarily caused by specific chemicals [13].

The coastal zone of Egypt on the Mediterranean extends over 1200 km from Rafah to El-Salloum [14]. Unfortunately, most of the Egyptian coastal zones along the Mediterranean Sea are subjected to intense discharges of pollutants from numerous anthropogenic activities [15, 16]. Along the Mediterranean coast of Egypt, there are eight coastal governorates. These are from west to east Matruh, Alexandria, Behaira, Kafr El-Sheikh, Damietta, Daqahliya, Port Said, and North Sinai. The enormous urban population and adjacent agricultural areas, all contribute to the pollution load reaching coastal waters. These derived either directly from coastal cities discharge points; the Rosetta branch of the River Nile, the Mahmudiya and Nubariya irrigation canals, drainage canals discharged directly to the sea, such as “El-Tabia and El- Ummum”, or from coastal lagoons “lakes” Maryut, Idku, Burullus and Manzala. Large parts of the Nile Delta suffer from severe coastal erosion, although adequate protection and mitigation measures have been considered. Most of the coastal lagoons “lakes” are however in crisis, suffering from the excessive discharge of industrial, agricultural and domestic sewage flow [17]. Alexandria governorate coastal zone receives a large amount of metal pollution from the principle industries of this region include fertilizers, agrochemicals, pulp, paper, power plant, food processing, detergents, fibres, dyestuffs, textile, and building materials where, the daily average industrial discharge amounts to 30,000 and 128–261,000 m^3^ per day domestic sewage and 1–2 million cubic meters per day of agricultural wastes [18]. Rashid, El-Gamil, Damietta and Port Said are exposed to agricultural drains contaminated with hazardous industrial wastes, domestic sewage, organic matter, fertilizers and pesticides, in addition to oil pollution from ships and oil terminal as in Port Said and Damietta [19].

The aim of the present study was to: (1) provide the concentration and distribution of some heavy metals in the Egyptian Mediterranean Sea sediments. (2) evaluate the potential ecological risk levels of some heavy metals by applying the Potential Risk Index Method. (3) investigate the biological effects of some heavy metals concentrations using available Sediment Quality Guidelines (SQGs); and (4) identify the sources of the heavy metals with multivariate analyses.

## Materials and methods

### Study area

**Fig. 1:**
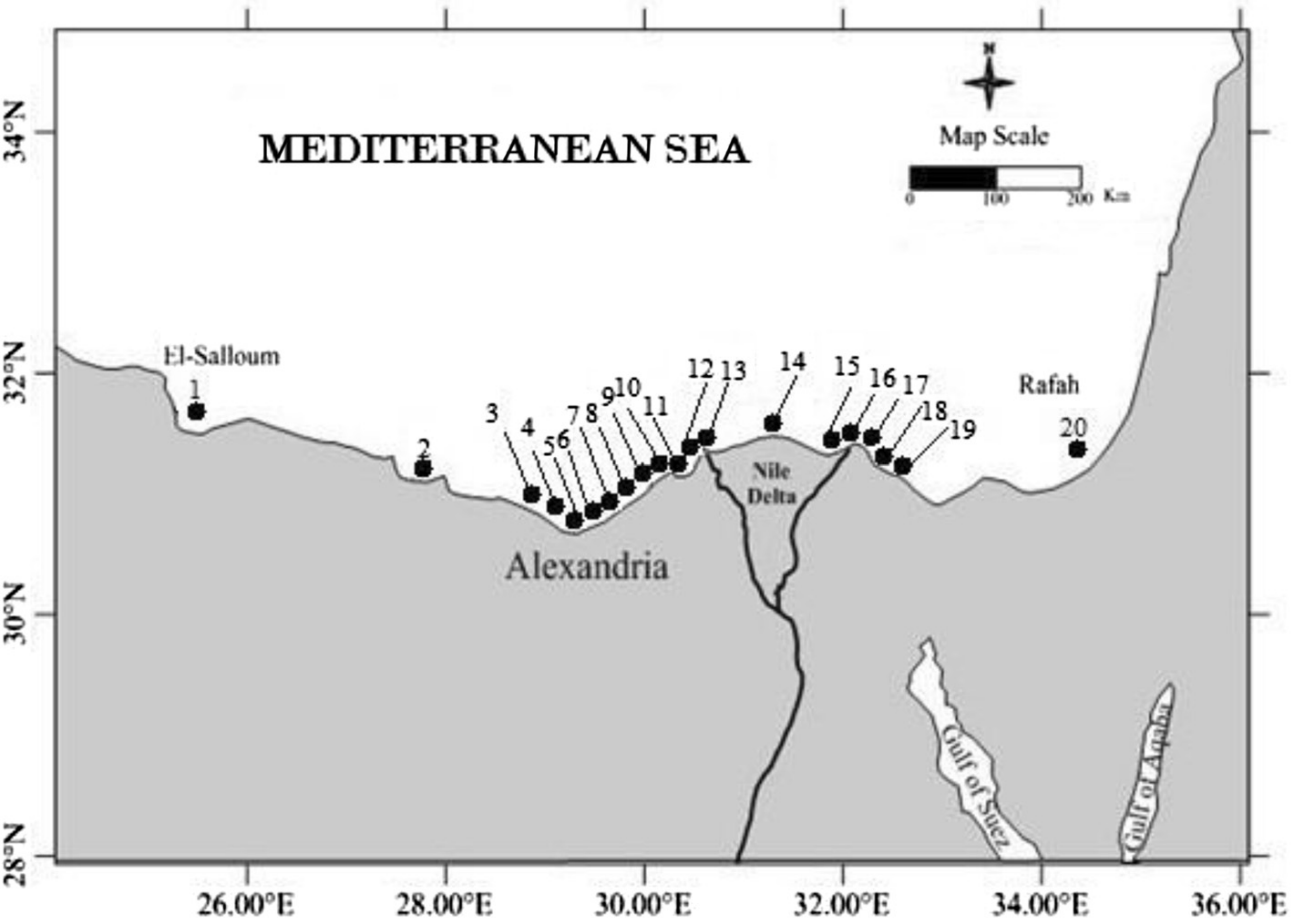
Study area and location of sediment sampling station

The Egyptian Mediterranean coast extends between longitude 25° 30′E and 34° 15′E and extends northward to latitude 33° N [20] (Fig. 1). Economic activities in the coastal zone include agriculture, industry, fishes/aquaculture, and recreation beaches. Half of Egypt’s industrial production comes from the delta, mainly from Alexandria. Main commercial ports are located at El Diekhila, Alexandria, Abu Quir, Idku, Damietta, Port Said, and east of Port Said [21].

### Sediment collection and pretreatment

Twenty surfacial sediment samples (0–5 cm depth) were collected during July 2010 from different selected stations along the Egyptian Mediterranean Sea using Peterson grab sampler (Fig. 1 and Table 1 ). The surface layer was chosen for this study, where this layer controls the exchange of metals between sediments and waters as well as constitutes a reserve of metals to which benthic organisms are exposed [20]. On the other hand, the sampling sites were selected to cover the expected polluted area due to industrial and other activities. Sufficient sediments had been collected from a particular site (six grabs). The samples were homogenized and placed into sealed polyethylene bags, carried to the laboratory in an ice box and stored at −20 °C in the dark until analysis.

### Analysis of sediment samples

For metal analysis, sediment samples were oven dried at 60 °C for almost a week. After drying, sediments were grounded to a size <63 μm in an agate mortar then stored in plastic vials until analysis. Then, the samples were digested in an open system with a mixture of concentrated HNO_3_/ HClO_4_/ HF (3:2:1) according to Oregioni and Aston [22]. The determination of the metals in the sediment samples were performed with a SHIMADZU AA6650, Kyoto, Japan atomic absorption spectrophotometer equipped with a deuterium background corrector. An atomizer with an air/acetylene burner was used for determining all the investigated elements. All instrumental settings were those recommended in the manufacturer’s manual book. Suitable internal chemical standards (Merck Chemicals, Germany) were used to calibrate the instrument.

Sediment pH was measured according to Yan et al. [23] with 1: 5 sediment to water ratio. Total organic carbon (TOC %) was determined according to Walkely-Blak’s wet oxidation method [24]. Total carbonate content of the sediment samples was determined by titration technique [25]. Grain size determination was made on the dried samples by the conventional sieving method [26].

### Quality control

To remove any contamination, all glassware and plastic vials were washed with 10 % nitric acid solution and rinsed thoroughly with Milli-Q water and dried. All reagents were Merck Chemiclas, Germany analytical grade or super pure quality. In order to check for the quality of the method applied for the analysis of heavy metals, the accuracy of the analytical method was estimated by analyzing sediment Standard Reference Material (IAEA-405): estuarine sediment, International Atomic Energy Agency, Vienna, Austria). Certified values of Fe, Mn, Zn, Cu, Cr, Ni, Co, Pb, and Cd are 37400, 495, 279, 47.7, 84, 32.5, 13.7, 74.8, and 0.73 μgg^−1^, and their measured values are 38334, 460, 252, 47, 78, 31, 14, 77, and 0.70 μgg^−1^, respectively. The recovery of the selected elements ranged from 90 to 104 % and the measurements of precision was under 10 % RSD. The detection limits of the instrument for each metal were 0.023, 0.033, 0.036, 0.047, 0.039, 0.040, 0.037, 0.044, and 0.025 mg/l for Cd, Cr, Cu, Co, Fe, Mn, Ni, Pb, and Zn, respectively.

### Assessment of ecological risk

#### Sediment quality guidelines

Numerous sediment quality guidelines are used to protect aquatic biota from the harmful and toxic effects related with sediment bound contaminants [27]. These guidelines evaluate the degree to which the sediment-associated chemical status might adversely affect aquatic organisms and are designed for the interpretation of sediment quality. They are also used to rank and prioritize contaminated areas for further investigation [28]. The National Standard of China (NSC) GB18668-2002 [29] has defined three grades of marine sediment, in which the content of some heavy metals is regarded as parameters used to classify marine sediments quality (Table 2). According to this criterion, the first class quality is suitable for mariculture, nature reserves, and endangered species reserves, and leisure activities such as swimming; the second class quality can be used for industry and tourism sites; and the third class can only be used for harbors.

**Table 1:** Physicochemical characteristics in sediments from the Egyptian Mediterranean coast

location	Station	CaCO_3_ %	TOC%	pH	Grain size analysis		
					Sand	Mud (Silt & clay)	Texture
El-Salloum	1	34.79	0.26	8.26	86.85	13.15	Very fine sand
Baghoush	2	94.45	0.32	8.21	99.99	0.01	Fine sand
El-Nobarreya	3	95.49	0.72	8.26	100	0.0	Fine sand
El-Dikhaila	4	85.42	0.34	8.31	99.96	0.04	Fine sand
El-Mex	5	67.24	0.31	7.98	100	0.0	Coarse sand
Western Harbour	6	95.57	0.38	7.82	100	0.0	Very coarse sand
NIOF	7	91.64	1.00	7.84	98.26	1.74	Fine sand
Eastern Harbour	8	93.56	0.57	8.38	99.99	0.01	Fine sand
Abu Qir	9	22.11	0.22	8.19	99.98	0.02	Fine sand
Power station	10	14.69	0.78	7.80	99.89	0.11	Fine sand
Maadia	11	15.64	0.28	8.02	99.96	0.04	Fine sand
Rashid West	12	3.83	0.05	7.93	100	0.0	Fine sand
Rashid East	13	2.85	0.11	7.41	99.93	0.07	Fine sand
Burullus	14	5.09	0.18	7.20	99.93	0.07	Fine sand
New Damietta	15	6.08	0.29	7.97	99.67	0.33	Very Fine sand
Ras El-Barr	16	4.87	0.14	7.63	99.57	0.43	Very Fine sand
El-Gamil West	17	4.85	0.19	7.56	99.72	0.28	Very Fine sand
El-Gamil East	18	5.64	0.20	7.77	99.76	0.24	Very Fine sand
Port Said	19	7.32	0.14	7.48	99.60	0.40	Very Fine sand
Rafah	20	6.33	0.20	7.79	100	0.0	Fine sand

**Table 2:** Total metals concentration (μgg^−1^ dry weight) in sediments from the Egyptian Mediterranean coast

	Cd	Co	Cr	Cu	Fe	Mn	Ni	Pb	Zn
Minimum	0.04	0.43	4.08	0.46	243	17	1.65	3.34	2.05
Maximum	0.47	26.39	297.95	26.26	38045	1086	60.25	53.67	62.21
Mean	0.22	8.24	82.74	8.46	13256	381	25.93	13.17	22.19
Median	0.17	4.47	53.1	7.32	7597	313.5	18.63	8.35	19.21
SD	0.15	8.40	90.18	6.22	12911	305	20.96	11.90	15.84
CV %	69	102	109	74	97	80	81	90	71
TEL	0.68	-	52.3	18.6	-	-	15.9	30.2	124
PEL	4.2	-	160	108	-	-	42.8	112	271
ERL	1.2	-	81	34	-	-	21	47	150
ERM	9.6	-	370	270	-	-	52	220	410
SEPA									
Class I	0.5	-	-	35	-	-	-	60	150
Class II	1.5	-	-	100	-	-	-	130	350
Class III	5	-	-	200	-	-	-	250	600
EPD									
Class A	<0.1	-	<25	<10	-	-	<15	<25	<70
Class B	0.1–1.0	-	25–50	10–41	-	-	15–35	25–65	70–150
Class C	1–1.5	-	50–80	55–64	-	-	35–40	65–75	150–200
Class D	>1.5	-	>80	>64	-	-	>1.5	>75	>200

*SD* Standard deviation, *CV* Coefficients of variation, *TEL* Threshold Effect Level, *PEL* Probable Effect Level, *ERL* Effects rang low, *ERM* Effects range median

Another classification system is the Hong Kong environmental Protection Department (EPD) [30] Classification system. In this system 4 classes are used to classify the sediment quality. The first class showed to be classified as uncontaminated sediment (Class A). Whereas, the second class represented (Class B) slightly contaminated sediment. The third and the fourth class were considered as moderately and seriously contaminated (Class C and Class D), respectively (Table 2).

U.S. National Oceanic Atmospheric Administration has developed Sediment Quality Guidelines (SQGs) for the assessment of sediment quality from the concentrations of contaminants using chemical and biological effects database [31]. The chemical concentrations corresponding to the 10th and 50th percentiles of adverse biological effects were called the effects-range-low (ERL) and ERM guidelines, respectively [31]. Another sediment quality guideline which is most widely used to assess the ecotoxicology of sediments is the TEL and PEL approach. This approach is based on the relation between measured concentrations of metals and observed biological effects, such as mortality, growth or reproduction of living organisms. Threshold effect level (TEL) refers to the concentration below which adverse effects are expected to occur only rarely and probable effect level (PEL) indicates the concentration above which adverse effects are expected to occur frequently occur [32].

### Mean PEL and ERM quotient

Although background/reference concentrations do give a base to evaluate SQGs and are important in environmental studies, they provide little insight into the potential ecological impact of contaminants [33]. Based on the fact that heavy metals always occur in sediments as complex mixtures, the mean PEL and ERM quotient method has been applied to determine the possible biological effect of combined toxicant groups by calculating mean quotients for a large range of contaminants using the following equation:

$$ \boldsymbol{E}\boldsymbol{R}\boldsymbol{M}\boldsymbol{\hbox{-}}\boldsymbol{Q}\ \boldsymbol{or}\ \boldsymbol{P}\boldsymbol{E}\boldsymbol{L}\boldsymbol{\hbox{-}}\boldsymbol{Q} = {\displaystyle \sum \left[{\boldsymbol{C}}_{\boldsymbol{i}}/\left(\boldsymbol{E}\boldsymbol{R}{\boldsymbol{M}}_{\boldsymbol{i}}\boldsymbol{or}\ \boldsymbol{P}\boldsymbol{E}{\boldsymbol{L}}_{\boldsymbol{i}}\right)\right]}\ /\boldsymbol{n} $$

Where C_i_ is the concentration of element i in sediments, ERM_i_, PEL_i_ the guidelines values for the element i and n is the number of metals. Mean quotients are considered as useful tools for reducing a large amount of contaminants into a single number. By calculating mean quotients it is assumed that adverse effects to marine organisms caused by individual chemicals are additional limitation is that this approach does not consider all the chemicals present in sediments but only those include in the SQG list [34]. Mean quotients can be used to identify, delineate and prioritize areas of potential concern with respect to quality of sediments [35]. ERMQ values of <0.1, 0.11–0.5, 0.5–1.5 and >1.5 related to 12 %, 30 %, 46 % and 74 % likehood, respectively, that sediments present toxicity in amphipod survival bioassays. Similarly, PELQ values of <0.1, 0.11–1.5, 1.51–2.3 and >2.3 coincide with 10 %, 25.5, 50 % and 76 % likehood of toxicity, respectively [31]. Consequently, four relative levels of priority (highly toxic, medium toxic, slightly toxic and non toxic) have been proposed.

### Potential ecological risk index method

The assessment of the potential risk of the heavy metal contamination was proposed as a diagnostic tool for water pollution control purposes as a result of the increasing content of heavy metals in sediments and their subsequent release into the water, which could threaten ecological health [36]. Potential ecological risk index method advanced by Swedish scholar Hakanson, according to the characteristics of heavy metal and its environmental behavior, is an approach to evaluate the heavy metal contamination from the perspective of sedimentology. It not only considers heavy metal level in the soil, but also associates ecological and environmental effects with toxicology, and evaluates pollution using comparable and equivalent property index grading method [37]. According to this method, the potential ecological risk coefficient E^i^_r_ of a single element and the potential ecological risk index RI of the multielement can be computed via the following equations:

$$ {\displaystyle {C}_f^i}={\displaystyle {C}_s^i}/{\displaystyle {C}_n^i} $$

$$ {\displaystyle {E}_r^i}={\displaystyle {T}_r^i}x{\displaystyle {C}_f^i} $$

$$ RI={\displaystyle \sum_{i=1}^n{\displaystyle {E}_r^i}} $$

where C^i^_f_ is the pollution coefficient of a single element of “i”; C^i^_s_ is the measured level of sedimentary heavy metal; C^i^_n_ is the background level of sedimentary heavy metal;T^i^_r_ is the toxic response factor for the given element of “i”, which accounts for the toxic requirement and the sensitivity requirement. The toxic response factors for Pb, Cd, Cr, Cu, Zn and Ni and Mn were 5, 30, 2, 5, 1, 5 and 1, respectively [11, 38]. Average shale values [39] and average crustal abundance [40] were commonly used to provide elemental background concentrations [41]. The average shale background concentration of global sediments [39] is selected as the reference baselines in this study.

According to Hakanson [11], the following terminologies are suggested for the E_r_ and RI values: (1) E_r_ <40, low ecological risk; 40 < E_r_ ≤80, moderate ecological risk; 80 < E_r_ ≤160, appreciable ecological risk; 160 < E_r_ ≤320, high ecological risk; and >320, serious ecological risk; (2) RI <150, low ecological risk; 150 < RI <300, moderate ecological risk; 300 < RI <600, high ecological risk; and RI ≥ 600, significantly high ecological risk.

RI method covers a variety of researching domains, i.e., biological toxicology, environmental chemistry as well as ecology, and can evaluate ecological risks caused by heavy metals comprehensively [11].

### Statistical analysis

Statistical methods were applied to process the analytical data in terms of its distribution and correlation among the studied parameters. MINITAB (version 14) package software was used for statistical analyses of the metal data. Basic statistical parameters such as range, mean, median, standard deviation (SD), and skewness were computed along with correlation analysis, while multivariate statistics in terms of principal component analysis (PCA) and cluster analysis (CA) were also carried out.

## Results and discussion

### Heavy metals in sediment

**Fig. 2:**
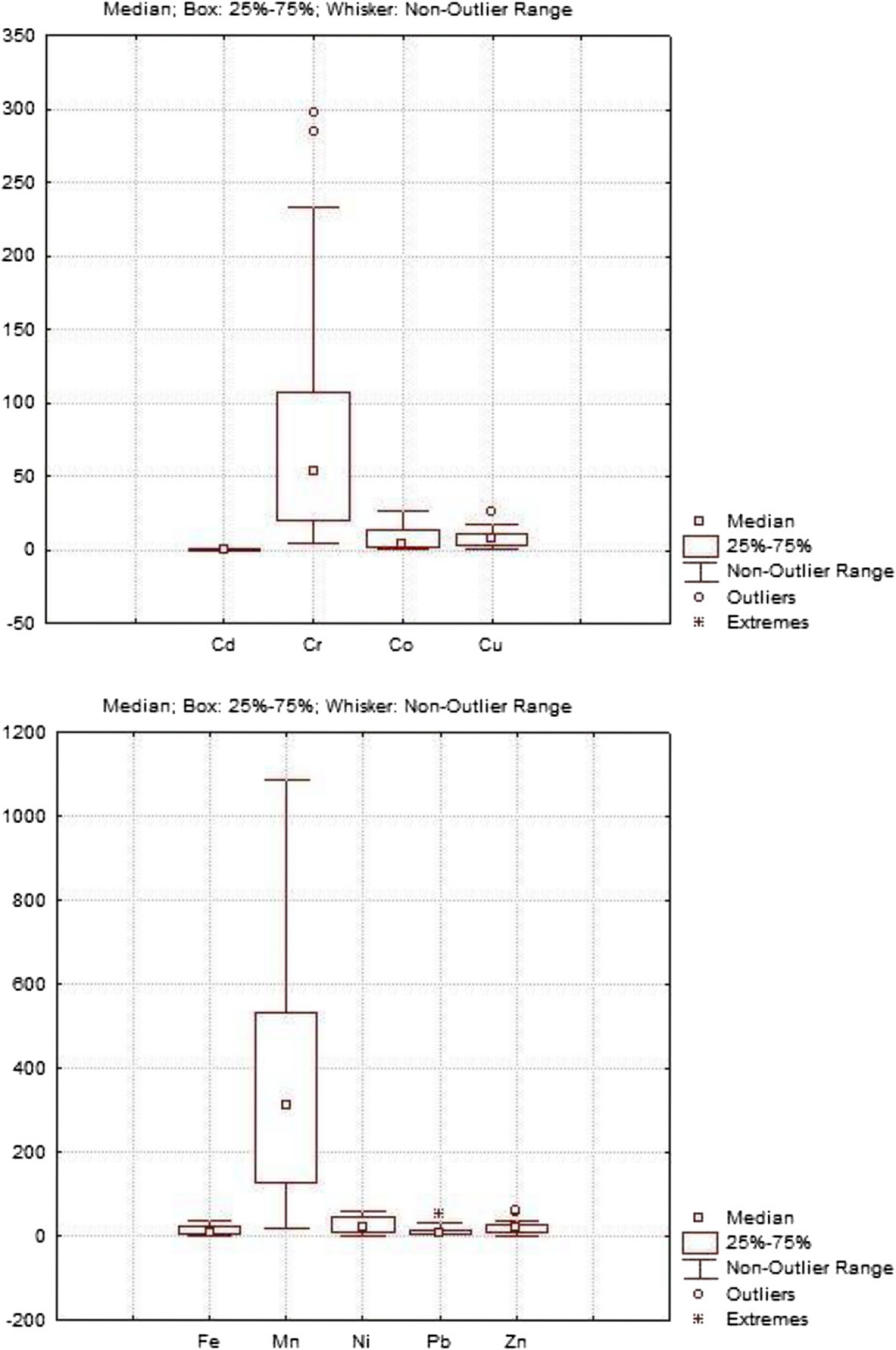
Concentrations of heavy metals (in μgg^−1^ except Fe in mgg^−1^) plotted in box and Whisker method

Figure 2 shows the results of analyses of heavy metals in sediment samples in box and whisker plot. Statistical summary of the metal contents including the mean value, background value, standard deviation and variation coefficients are presented in Table 2. Among the 9 elements studied, concentrations of Fe and Mn were higher, whereas lower concentrations of Co and Cd were observed in the different sampling locations.

In general, the results obtained in this study were in the range observed in other Mediterranean countries (Table 3), or even lower in some cases. In this sense, cadmium and lead showed higher concentrations in Libyan Mediterranean coast. Whereas, Co, Mn and Ni showed concentrations lower than those reported in Ivra complex, Italy. On the other hand, the values of Cd, Cr, Cu, Co, Ni, and Pb obtained at the Moroccan Mediterranean coast were very close to those obtained in this study. However, the Cr concentrations were higher in the Egyptian Mediterranean coast than those measured in Malaga Bay, and the Libyan Mediterranean coast.

**Table 3:** Comparison between heavy metals concentrations obtained in this study with those obtained by other authors in Mediterranean Sea

Location	Cd	Cr	Cu	Co	Mn	Ni	Pb	Zn	Reference
Mediterranean Sea Egypt	0.04–0.47	4.08–297.95	0.46–26.26	0.43–26.39	17–1086	1.65–60.25	3.34–53.67	2.05–62.21	Present study
Malaga bay Mediterranean Sea Spain	0.021–0.283	4.31–26	6.57–21.2	-	-	9.48–40.2	7.92–37.1	-	[47]
Mediterranean Sea Morocco	0.14–0.27	88.40–160.97	4.09–29.12	18.06–31.7	256.56–651.66	3.19–79.89	33.11–47.97	64.82–110.77	[48]
Mediterranean Sea Turkey	-	-	-	-	283–1192	28–240	91.3–751	86–970	[49]
Ivra Complex Italy	-	2568–2984	-	100–117	968–1053	2040–2438	0.25	-	[50]
Mediterranean Sea Libya	5–10.5	14.8–24.9	9.1–22.7	8.2–18.1	14.3–49.4	11.6–29.9	8.9–56.9	11.6–30.5	[51]
Eastern Mediterranean Sea Egypt	1.8–2.3	-	4–9.4	-	200.8–254.3	-	18.4–24.8	33.1–42.2	[52]
Average shale	0.3	90	45	19	850	68	20	95	[39]

In order to establish relationships among metals and determine the common source of metals in the Egyptian Mediterranean coast, a correlation matrix was calculated for heavy metals in the sediments. The data showed that strong positive correlation exists between Fe and Mn (*r* = 0.92, *p* < 0.01), Co (*r* = 0.96, *p* < 0.01), Cr (*r* = 0.69, *p* < 0.01), and Ni (*r* = 0.85, *p* < 0.01). It means that these metals tend to accumulate together. The significantly positive correlation with Fe indicates that the metals were derived from similar sources and also moving together [42]. Significant correlation exists between Zn and Cu (*r* = 0.80, *p* < 0.01) and Pb (*r* = 0.66, *p* < 0.01), which suggested that these metals were redistributed in the sediments by the same physico-chemical processes or had a similar source [3]. The minor role of carbonate as metal carrier is reflected by the negative correlations between Ni (*r* = − 0.81, *p* < 0.01), Fe (*r* = − 0.68, *p* < 0.01), Mn (*r* = − 0.68, *p* < 0.01), Co (*r* = − 0.69, *p* < 0.01) and Cr (*r* = − 0.65, *p* < 0.01) with CaCO_3_. On the other hand, the negative correlation of TOC with Ni (*r* = − 0.57, *p* < 0.01), Fe (*r* = − 0.48, *p* < 0.05), Co (*r* = − 0.48, *p* < 0.05) and Cr (*r* = 0.53, *p* < 0.05) suggested that TOC doesn’t have important role in the binding of these elements [16].

### Assessment of ecological risk

#### Sediment quality guidelines

The sediments quality guidelines for the selected metals and a classification of the samples based on the guidelines are shown in Table 2. The contents of Cd, Cu, Pb and Zn at all samples are lower than the upper limit of the first class criteria of NSC GB 18668–2002. Comparing the sediment of the present study with classification system from Hong Kong environmental Protection Department (EPD) Classification system, the value of the mean Copper, Zinc, and Lead concentrations showed to be classified as uncontaminated sediment (Class A). Whereas, Nickel and Cadmium represented (Class B) contaminations. Sediments were considered as seriously contaminated (Class D) when comparing the mean concentration of Chromium with the classification system adopted by the Hong Kong Government [30].

Comparing results of the present study with ERL and ERM values, it was observed that Cd, Cu, and Zn at 100 % of sampling stations are below the ERL value (1.2, 34, and 150μgg^−1^), respectively which indicate that these metals are not likely to have adverse effects on animals that live in the sediment. Only one station (El-Mex) which had a Pb concentration > ERL, indicated that Pb at El-Mex will likely to has effects on animals that live in this sediment. On the other hand, all the rest of the studied station had a concentration of Pb below the ERL value which indicates that Pb in the study area is not likely to have adverse effects on animals that live in sediments except station 5 in El-Mex. On the other hand, Ni at 30 % of sampling stations (Rashid west, Burullus, New Damietta, El-Gamil east, Port Said and Rafah) had a value over the ERL value (36.384, 43.545, 44.305, 48.93, 47.415, and 27.79), respectively. This reflects that the adverse effects on animals live at these stations are frequently occurred. Stations 13, 16, and 17 in (Rashid east, Ras El-Barr and El-Gamil west), respectively had concentration of Ni above the ERM value (56.536, 56.413, and 60.246) which means that Ni probably has adverse effects on animals live in this sediment.

When compared to the TEL-PEL SQGs, the concentrations of Cd and Zn are lower than the TEL value at 100 % of sampling stations, while Pb and Cu showed values lower than the TEL at 95 % of sampling stations. On the other hand, in case of Ni, 20 % of samples fall in the range between TEL and PEL at Abu Qir, Electric power station, Rashid west and Rafah indicating associated adverse biological effects may occasionally occur. However, exceedance of SQG values does not firmly guarantee the occurrence of deleterious ecological effects, unless they are also coherent with regional background levels [43]. Although about 35 % of sediment samples had concentrations of Ni exceeding their respective PEL values at Rashid east, Ras El-Barr, New Damietta, Burrllus, El-Gamil east and west and Port Said, which were expected to have adverse biological effects occasionally, however, 100 % of sediment samples had the concentration of Ni lower than their respective Background levels (68 mg/kg) of average shale [39]. Furthermore, Cr exceeds the PEL value at 15 % of samples (Rashid east, El-Gamil west and Port Said).

### Mean PEL and ERM quotient

**Fig. 3:**
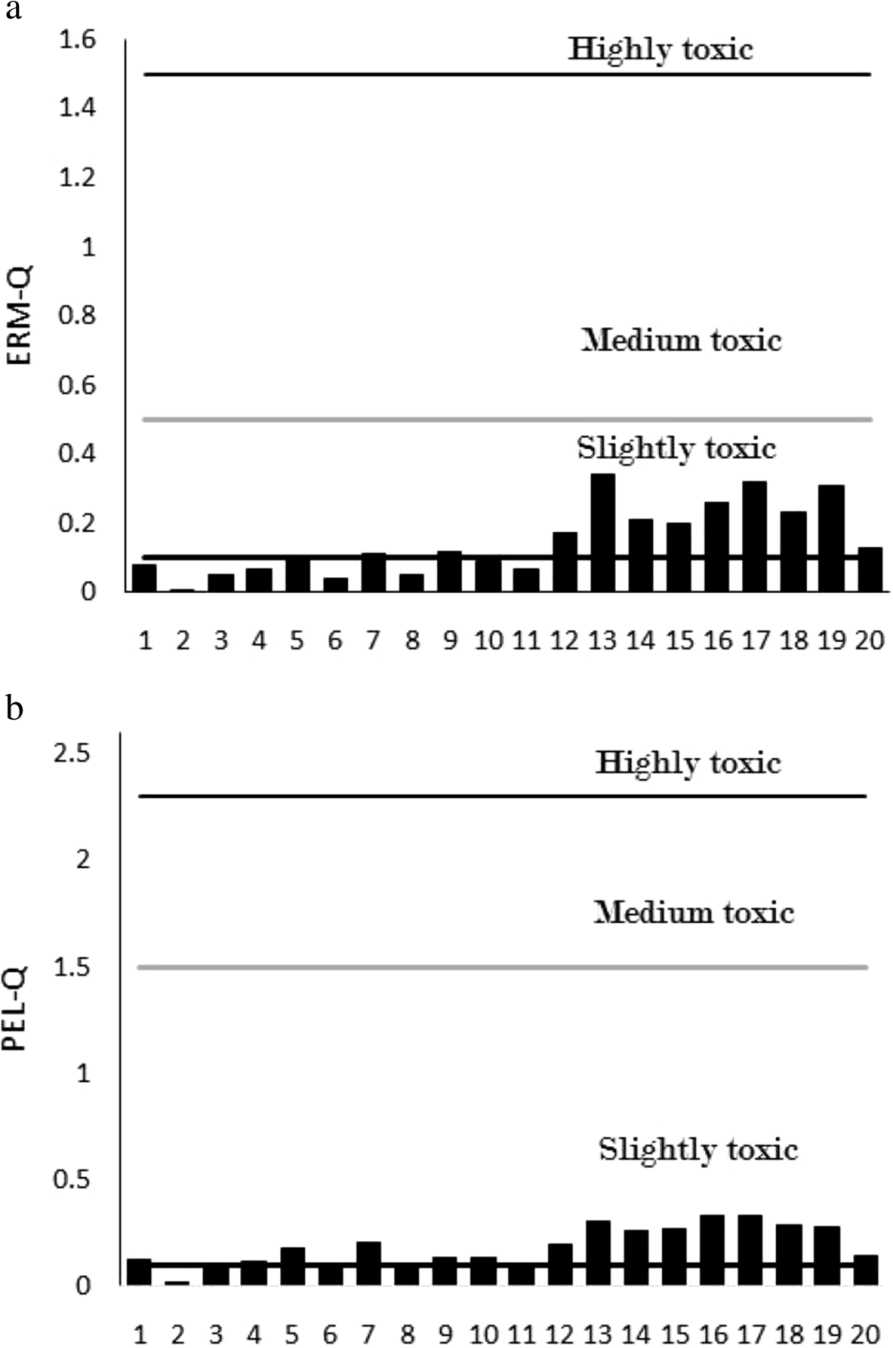
**a** Estimated mean ERM-Q of surface sediments from the Egyptian Mediterranean coast. **b** Estimated mean PEL- Q of surface sediments from the Egyptian Mediterranean coast

**Fig. 4:**
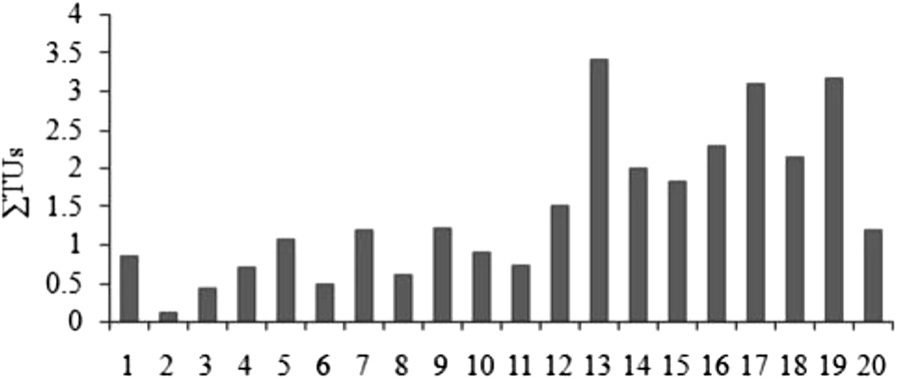
Estimated sum of the toxic units (∑TUs) of surface sediments from the Egyptian Mediterranean coast

The *m- ERM*-Q calculated for the sampling sites (based on metals Cd, Cr, Cu, Ni, Pb and Zn) ranged from 0.01 to 0.34 (mean value of 0.15) (Fig. 3a), indicating that the combination of Cd, Cr, Cu, Ni, Pb and Zn may have a 30 % probability of being toxic. Only eight stations (40 %) have ERMQ (<0.1) and are categorized as non toxic and the rest of stations are categorized as slightly toxic. On the other hand, the *m-PEL–Q* in surface sediments of the Egyptian Mediterranean coast range from 0.02 to 0.57 (mean value of 0.24) (Fig. 3b), indicating that the combination of Cd, Cr, Cu, Ni, Pb and Zn may have a 25 % probability of being toxic. Furthermore, potential acute toxicity of contaminants in sediment samples could be estimated as the sum of the toxic units (∑TUs) defined as the ratio of the determined concentration to PEL value [44]. In Fig. 4, the values of sum of TUs for each sampling stations based on the concentrations of Cd, Cr, Co, Cu, Fe, Mn, Ni, Pb and Zn were shown. The sum of the toxic unit at Rashid east, El-Gamil west, and Port Said exhibit higher levels than other stations.

### Potential ecological risk index method

To confirm the above evaluation, we further calculated the potential ecological risk index (RI) of surface sediments from the Egyptian Mediterranean coast. The results of evaluation on potential ecological risk factor (E^i^_r_) and the potential ecological risk index (RI) are summarized in Table 4. The order of potential ecological risk coefficient (E^i^_r_) of heavy metals in sediments of the Egyptian Mediterranean coast was Cd > Pb > Ni > Cr > Cu > Mn > Zn. The mean potential ecological risk coefficient of Cd, Cr, Cu, Mn, Ni, Pb and Zn were all lower than 40, which belong to low ecological risk. All the sampling sites were at low risk level where the RI values were much lower than 150.

**Table 4:** Evaluation on potential risk of heavy metals pollution in sediments from the Egyptian Mediterranean coast

Station	Potential ecological risk factor E^i^_r_							RI	Risk grade
	Cd	Cr	Cu	Mn	Ni	Pb	Zn		
1	28.70	0.70	1.54	0.14	0.56	4.09	0.41	36.13	Low
2	7.40	0.09	0.02	0.03	0.12	0.90	0.02	8.58	Low
3	6.40	0.12	0.10	0.02	0.70	3.67	0.10	11.10	Low
4	34.60	0.36	1.04	0.17	0.64	3.07	0.24	40.13	Low
5	16.10	0.52	0.82	0.12	0.42	13.23	0.52	31.72	Low
6	46.90	0.16	0.19	0.27	0.27	1.93	0.07	49.79	Low
7	44.10	0.56	2.88	0.18	0.64	7.48	0.65	56.50	Low
8	38.30	0.21	1.22	0.16	0.20	6.11	0.21	46.41	Low
9	19.00	1.90	0.63	0.62	1.42	2.36	0.17	26.11	Low
10	39.10	0.75	0.26	0.49	1.32	1.46	0.11	43.48	Low
11	44.70	0.68	0.55	0.43	0.84	1.73	0.15	49.09	Low
12	14.20	1.87	0.35	0.27	2.68	1.15	0.11	20.62	Low
13	9.20	6.62	0.78	0.93	4.16	1.60	0.26	23.56	Low
14	23.20	2.60	1.10	0.63	3.20	1.45	0.18	32.36	Low
15	3.50	1.76	1.33	0.97	3.26	2.21	0.28	13.30	Low
16	5.00	2.27	1.92	0.85	4.15	1.74	0.31	16.24	Low
17	14.90	5.19	0.78	0.57	4.43	1.61	0.19	27.67	Low
18	12.60	2.49	1.31	0.57	3.60	1.97	0.28	22.81	Low
19	4.30	6.32	1.27	1.28	3.49	1.67	0.32	18.63	Low
20	18.10	1.61	0.17	0.06	2.04	0.83	0.03	22.85	Low
Mean	21.52	1.84	0.91	0.44	1.91	3.01	0.23	29.85	Low

### Multivariate statistical analysis

Multivariate analysis (i.e., Principal component analysis; PCA and Cluster analysis; CA) has been proved to be an effective tool for providing suggestive information regarding heavy metal sources and pathways [45].

**Fig. 5:**
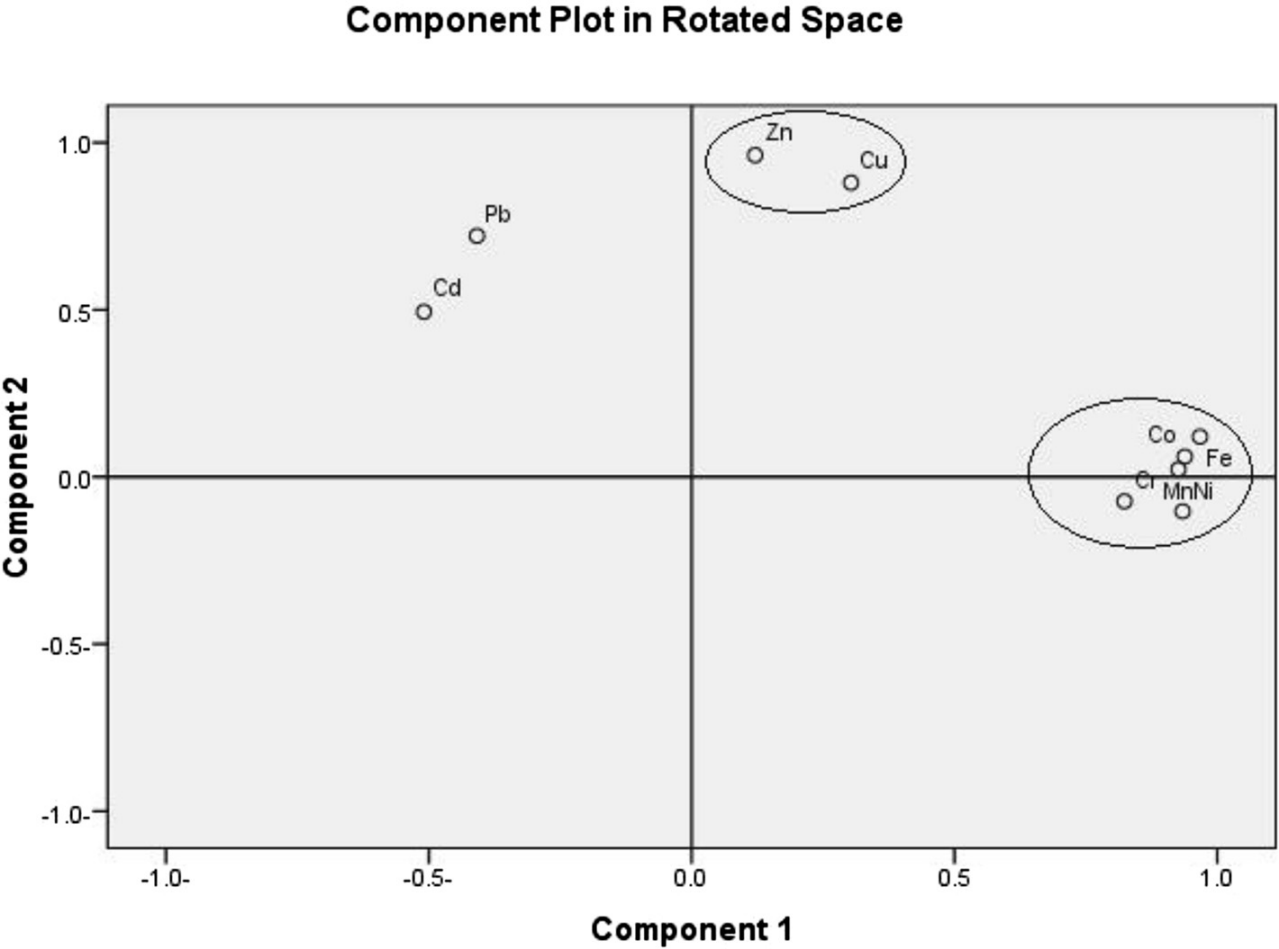
Loading plot of heavy metals in the space defined by PC1 and PC2

The results of the principal component analysis; PCA on the data matrix obtained from total metal analysis of surface sediments along the study area are shown in Table 5. Two main components with Eigenvalues greater than 1 were determined, explaining 80.14 % of the total variance. Apparently the result of PCA corresponds well with the correlation coefficients. The first component (PC1), with a variance of 55.059 %, was highly correlated with Ni, Fe, Co, Mn and Cr; correlation coefficients among this group of elements exceed 0.7 (0.945, 0.953, 0.924, 0.911 and 0.833, respectively). On the other hand, cadmium and lead showed strong negative loading (−0.648 and −0.542). Co, Ni and Cr belong to the siderophile elements, and are main rocks forming elements. It is easy for them to enter into iron magnesium silicate minerals, because of their similar ionic radius. This element association is considered to represent the lithology of the study area, and a natural input, i.e., they are derived from terrigenous detritus material transported by surface runoff [46]. The second component (PC2) explained 25.11 % of the total variance with significant loadings on Zn and Cu (0.966 and 0.876 respectively), which suggests similar sources. However, Pb also showed moderate positive loading (0.669), suggesting that the sources of Pb could be both natural and anthropogenic. Cadmium displays none of strong correlations between the other metals, suggesting that Cd has another different sources or pathways [45]. PC1 and PC2 together explained 80.14 % of the total variance, indicating that the lithogenic factor dominates the distribution of most part of the considered metals in the study (Fig. 5).

**Table 5:** Factor loadings on elements in sediments from the Egyptian Mediterranean coasta (*n* = 20)

Element	PC1	PC2
Zn	0.037	0.966
Ni	0.945	-.052
Pb	−0.542	0.669
Cd	−0.648	0.209
Fe	0.953	0.190
Cu	0.229	0.876
Mn	0.911	0.110
Co	0.929	0.129
Cr	0.833	−0.032
Eigenvalue	4.955	2.260
% variance explained	55.059	25.115
Cumulative % variance	55.059	80.174

Extraction method: Principal component analysis

Rotation method: Varimax with Kaiser Normalization

**Fig. 6:**
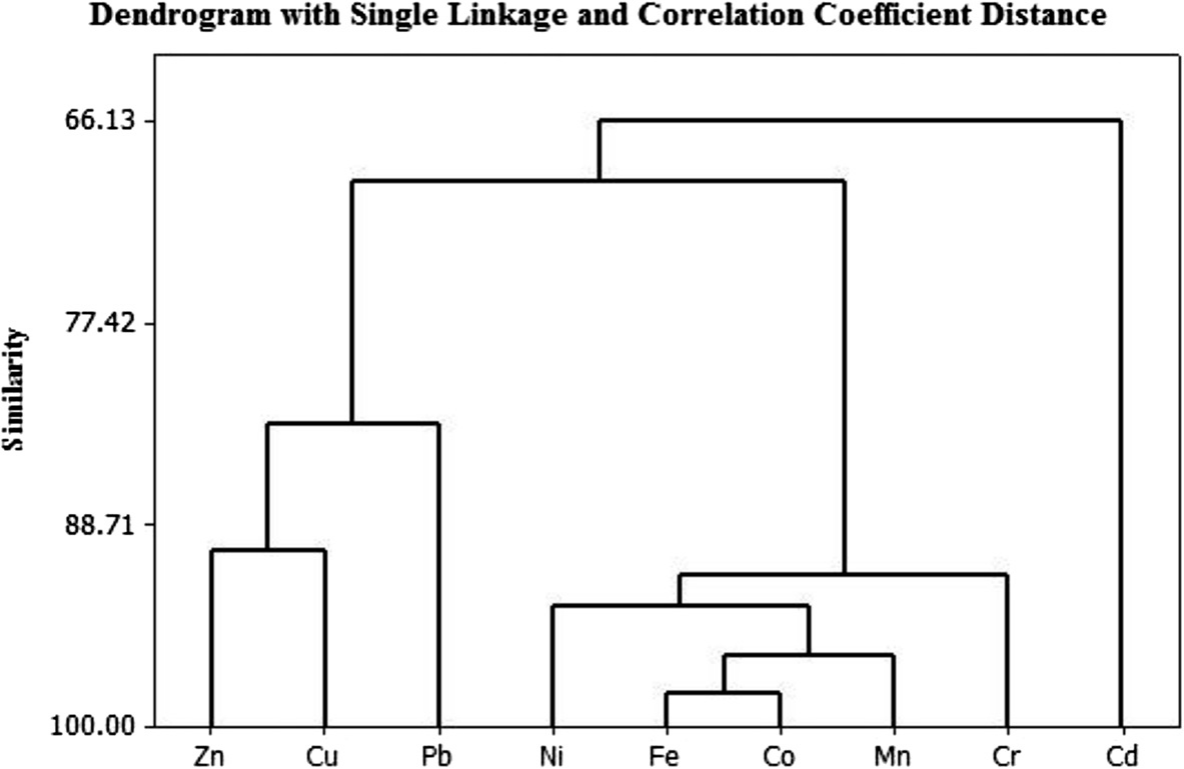
Dendrogram showing cluster of variables on the basis of similarity

Cluster analysis is often coupled with PCA to confirm results and provide grouping of variables [45]. In this study, CA was performed on the same data as PCA to understand the similarities among them. Figure 6 depicts a dendrogram with single linkage Euclidean and correlation coefficient distance. The cluster analysis results indicate two clusters: (1) Pb-Zn-Cu; (2) Ni-Mn-Fe-Co-Cr in terms of similarities. This indicates that Ni, Mn, Fe, Co, and Cr appear to have originated mainly from natural sources. In addition, Pb, Zn and Cu seem to drive partly from sources other than Ni, Mn, Fe, Co and Cr. This is consistent with our PCA results.

## Conclusion

The results of this study provide valuable information about metal contamination in sediments along the Mediterranean Sea from El-Salloum to Rafah for over than 1200 km. The distribution pattern of heavy metals in the sediments followed the sequence: Fe > Mn > Cr > Ni > Zn > Pb > Cu > Co > Cd. Association with adverse biological effects to aquatic biota was also assessed using the classification of sediments and sediment quality Guidelines (SQGs). Ni and Cr exceeded the probable effect level (PEL) in 35 and 15 % of the sampling sites, respectively. The ecotoxicological index expressed as the mean ERM quotients (m-ERM-Q) suggested that the combination of Cd, Cr, Cu, Ni, Pb and Zn may have a 30 % probability of being toxic while, the (m-PEL-Q) showed only a 25 % probability of being toxic due to the combination of these metals. Similar results are also obtained by the potential ecological RI, with the average Er for heavy metals decreased in the order: Cd > Pb > Ni > Cr > Cu > Mn > Zn. Multivariate statistical analysis evidenced significant correlations between Fe, Mn, Co, Cr and Ni, suggesting similar sources and/or similar geochemical processes controlling the occurrence of these metals in the sediments. This study supports metal pollution monitoring and control for the Egyptian Mediterranean Sea. It will be a useful tool to authorities in charge of sustainable marine management.
